# Supplementary material for: Are carbon emissions trading and green financial instruments synergistic? -Comprehensive quantitative research based on content analysis
Source: PLoS One. 2024 Mar 7;19(3):e0298601. doi: 10.1371/journal.pone.0298601 (PMC10919601; doi:10.1371/journal.pone.0298601)
Supplement: S1 Data — (ZIP) [file pone.0298601.s001.zip › Data/地级市Z_F工作报告.zip!/║╙─╧/░▓╤⌠╩╨/└┤╫╘╣1⁄2╓┌║┼í╛┬φ┐╦╩2╛▌═°í┐_2008.docx]

各位代表:

现在，我代表市人民政府，向大会作政府工作报告，请予审议，并请各位政协委员提出意见。

一、2007年工作回顾

2007年，市人民政府在省委、省政府和市委的正确领导下，紧紧围绕"一强五前"和构建和谐安阳的奋斗目标，全面落实科学发展观，积极顺应宏观调控，扎实工作，创优争先，较好地完成了市十一届人大四次会议确定的各项目标任务。

--总体实力再上新台阶。全市生产总值808亿元，增长17.9%，高于全省3.5个百分点，创14年来最高水平。人均生产总值15530元，增长18.1%，高于全省3.6个百分点。

--质量效益得到新提升。全市限额以上工业企业实现利润127.3亿元，增长100.1%，高于全省31.6个百分点。经济效益综合指数287.2，提高67.3点;财政一般预算收入42.5亿元，增长22.8%;一般预算支出80.4亿元，增长26.4%，首次高于一般预算收入增幅。

--工业、农业取得新发展。全市限额以上工业增加值360亿元，增长27.5%，增速居全省第二，连续5年保持20%以上的高速增长。全年粮食总产325.4万吨，增长10.9%，连续4年实现丰产丰收。

--投资、消费和进出口实现新突破。全社会固定资产投资429.5亿元，增长38.5%，高于全省2.9个百分点。全社会消费品零售总额198.6亿元，增长18.4%，增速居全省第五。进出口总值13亿美元，增长68.9%;出口完成6.3亿美元，增长67.2%，增速居全省第一。

--人民生活水平有了新改善。城镇居民人均可支配收入11796元，增长17.9%，高于全省0.9个百分点;农民人均纯收入4493元，增长19.1%，高于全省1个百分点，连续4年保持两位数增长。居民储蓄存款余额442.7亿元，增长5.6%。

一年来，我们主要做了以下工作:

(一)积极优化工业结构。突出工业的主导地位，产业竞争力明显增强。"工业振兴工程"强力推进。强化了市领导联系项目制度和工业振兴工程现场推进会制度，从领导力量、政策措施、协调服务、资金投向等方面培育做强重点企业，促进了经济总量大幅提升。加快98个重点项目建设，安钢"三步走"战略基本完成，成为产钢千万吨级现代化钢铁集团，安烟技改扩建工程、安化年产6万吨甲胺、昌泰纺织三期6.5万锭棉纱、宝硕化工年加工15万吨煤焦油、科能特钢双金属耐磨合金管道生产线等42个项目竣工投产。11户企业入围省百户重点企业和50户高成长性企业。全市销售收入超亿元的企业达166家，超10亿元的达21家。工业结构进一步优化升级。加大产业整合力度，推进产业集群发展，完善了百户重点企业发展规划，钢铁、煤炭等行业整合迈出实质性步伐。9家煤炭企业整合成鑫龙煤业集团，组建了鑫盛机械装备集团。企业自主创新能力得到加强。安钢集团技术中心被认定为国家级企业技术中心，有12家企业被认定为省级企业技术中心和工程技术中心。全市省级高新技术企业达61家，高新技术产品达178个。节能减排和环境保护成效明显。实施煤气回收发电、TRT压差发电等一批节能技改项目，循环经济试点顺利实施。安林两路环境综合整治稳步推进，在全省率先彻底淘汰水泥机立窑生产线23条，200立方米以下炼铁高炉正在加紧拆除。关停小火电机组60.8万千瓦，基本拆除粘土砖瓦窑厂。预计全市单位生产总值能耗下降4%左右，化学需氧量、二氧化硫排放量分别削减0.5307万吨和1.0686万吨。

(二)扎实做好"三农"工作。以建设农业产业化强市为目标，强力推进"四大兴农计划、三大惠民工程、十大产业链开发和十大农业增效技术推广"。农业产业化水平得到整体提升。全市较大规模的农业龙头企业达111家，实现利税10.3亿元，带动40万农户。汤阴县、滑县、安阳县农副产品加工基地区入驻企业48家。培植农业产业化和特色农村经济先进乡镇34个。建设优质高产粮食基地300万亩。高效农业和现代畜牧业发展势头强劲。新建百亩园、千亩方、万亩区56个，新增高效农业面积21万亩。新建畜禽养殖密集区85个，全市达到216个，新增大中型规模养殖企业87个。星河、健丰、众品、尚品等60多家龙头企业带动粮食、棉花、花生、畜禽、木材加工等十大农业产业链进一步拉长。农村生活条件得到明显改善。认真落实我市八个以奖代补文件，调动社会资金5.9亿元投向农村、农业。新增农村沼气用户4.4万户，解决了67万农民用气问题。筹措资金6844万元，解决农村安全饮水18万人。完成农村劳动力转移培训11.6万人。新建农村超市1056家。争取上级无偿资金2.8亿元，落实粮食直补、畜牧养殖补贴等各类补贴2.6亿元。新农村建设稳步推进。"十大标兵村"和130个试点村建设成效明显，乡村道路、饮水、沼气、电力、超市、村庄绿化等各项事业健康发展。

(三)切实加强基础能力建设。积极优化投资结构，加快建设了一批国家鼓励发展的重点项目。投资结构进一步优化。全市城镇固定资产投资356亿元，增长39.8%。符合国家产业政策的机械、电子等行业投资得到加强，比重分别增加1.7个和0.4个百分点;国家重点监测的冶金行业投资得到一定控制。重点项目建设积极推进。125项省、市重点建设项目累计完成投资114.3亿元，占年计划的100.8%。完成济东高速公路滑县段建设任务，安南高速进展顺利，安姚公路全线和大唐安阳电厂2×30万千瓦机组已开工建设，改造和新建农村公路410公里，南水北调安阳段和马家岩水库等项目顺利实施。城市建设和管理得到加强。城市总体规划修编纲要已通过建设部技术认定。圆满完成中华路开发区段、南出口改造工程等4条道路建设任务;华祥立交桥、铁西路改造和宗村污水处理厂、市医疗垃圾处理厂等项目顺利实施。5县(市)全部建成污水处理厂。洪水河综合改造工程开工建设。新增经济适用住房22.7万平方米、城市公共绿地281万平方米、城市集中供热55万平方米、天燃气用户1万户。加强城市管理，我市荣获2007年"中国城市管理进步奖"。新"四创"工作扎实推进。创建全国科技进步先进市和省文明城市取得成功，创建国家卫生城市正在艰苦努力，创建国家园林城市有序推进。

(四)强力推进改革开放。坚持把改革开放贯穿于发展的全过程，经济发展活力进一步增强。各项改革稳步推进。25户国有企业完成改制，沙钢、永煤、河南中孚、香港华润等公司通过增资扩股等形式与我市企业实现了战略重组。金融事业和改革取得进展，各项贷款余额392.8亿元，较年初净增58.3亿元。城市信用社被国家银监会批准筹建市商业银行。农村信用社完成票据兑付6.4亿元。邮政储蓄银行挂牌运行。农业银行股改确权工作积极推进。市信用担保公司增资扩股任务完成。科技、教育、文化、卫生、财政等领域改革也都迈出了新的步伐。企业上市稳步推进，鑫盛机床、豫北金铅等企业被列入我省今年重点推荐A股上市企业。非公有制经济快速发展。全年实现增加值391亿元，增长20%。开放型经济成效显著。组织参加了中部博览会、豫港经贸洽谈会、全球客家招商会、厦门国际投资贸易洽谈会等大型经贸活动，开展了赴广州、温州、青岛集中招商引资活动，举办了豫商安阳发展论坛、知名企业家安阳行、在外创业英才故乡行活动。全市实际利用外资7461万美元，增长56.9%;引进域外资金175亿元，增长42.6%。第三产业不断壮大。实现增加值205亿元，增长15.7%，为14年来最高。积极发展文化旅游、生态旅游和红色旅游，全年接待国内外游客1107万人次，旅游总收入40.6亿元，增长13.2%。大力发展金融、房地产、信息等新兴服务业和现代物流业，沃尔玛、大华、"八挂来网"等一批大型商贸和物流项目正在加快建设。县域经济实力明显提升。全市县域生产总值567亿元，增长20%;财政一般预算收入16.1亿元，增长27%，其中2个县(市)超5亿元。

(五)积极构建和谐安阳。着力解决人民群众最关心、最直接、最现实的利益问题，十个方面44件实事圆满完成。就业再就业工作取得明显成效。新增城镇就业人员12.3万人，安置下岗失业人员5.1万人，其中"4050"等困难群体1.9万人。在全省率先基本消除城镇零就业家庭，开展了农村零转移就业家庭帮扶活动。社会保障体系不断完善。社会保险覆盖面进一步扩大，养老、医疗等6项保险参保人数不断增加。城市低保对象9万人，发放低保金8579万元;农村低保对象13.9万人，低保标准由每人每月20元提高到30元，全年发放低保金4413万元。健全城镇低收入家庭廉租房制度。完成了45所敬老院改扩建工程，五保集中供养率达30.1%。较好地解决了3.03万农村贫困人口的脱贫和温饱问题。各项社会事业长足发展。4项科技成果荣获国家科技进步二等奖，6项科技成果荣获省科技进步奖，3个项目被批准为国家星火计划项目，成功举办了第六届全国特种养殖种植品种展示交流会。各级各类教育持续快速发展，农村办学条件明显改善。"两基"迎国检顺利通过验收。安阳师范学院以优异的成绩通过教育部本科教学水平评估。全市落实"两免一补"资金1.64亿元，惠及学生117万人次。中国文字博物馆开工建设，市图书馆、博物馆基本完工，推出了《大爱无言》等一批文艺精品。公共卫生和医疗服务体系进一步健全，初步建立起覆盖市区的社区卫生服务网络，新型农村合作医疗制度实现了全覆盖，参合农民387万人，参合率92.5%。深入开展全民健身活动，积极推进"农民体育健身工程"和社区体育工作，成功举办了市九运会。大力培养和引进人力资源，不断加强人才队伍建设。低生育水平继续保持稳定，全年人口自然增长率5.19‰，低于全国、全省水平。

(六)不断加强精神文明和民主法制建设。社会主义荣辱观教育和精神文明创建活动深入开展，涌现出谢延信、周国允、吴新芬、王生英等一批先进典型人物。坚持依法治市，严格依法行政，自觉接受人大法律监督、工作监督和政协民主监督，认真办理4件人大议案、326件人大代表建议和604件政协委员提案。安全生产工作成绩显著，被省政府评为完成安全目标优秀市，各类事故起数和死亡人数分别下降34.9%和23.2%。高度重视信访工作，在全国率先成立"和谐委"，信访"三级例会制度"被中央联席办、国家信访局总结推广。社会治安防控体系不断完善，社会大局保持稳定。双拥共建工作深入开展，军政军民团结进一步加强，第四次被命名为全国双拥模范城。切实纠正部门和行业不正之风，反腐倡廉工作取得新进展。不断加强老区建设，依法保护妇女儿童合法权益，老龄和关心下一代工作健康发展。民政、统计、审计、史志、档案、经济研究、外事侨务、民族宗教、无线电管理、红十字等工作也都取得了新成绩。

各位代表!

这些成绩的取得，是市委正确领导的结果，是市人大、市政协大力支持的结果，是全市人民同心同德、团结奋斗的结果。在此，我代表市人民政府，向奋斗在全市各条战线上的广大工人、农民、干部、知识分子、解放军指战员、武警官兵、政法干警以及各界人士，致以崇高的敬意!向所有关心、支持我市现代化建设的港澳台同胞、海外侨胞、国际友人，表示衷心的感谢!

在肯定成绩的同时，我们也清醒地认识到，我市的经济社会发展还存在着一些矛盾和问题。一是产业结构不尽合理，三产比重偏低，受国家调控的钢铁、水泥、冶炼行业比重过大，高新技术产业、高附加值产品不多。二是资源环境约束加剧，节能减排任务繁重。三是县域经济发展不平衡，部分县区缺乏支撑税源的大企业大项目，其财政困难状况没有大的改变。四是由于市场物价上涨，城乡低收入群体生活受到一定影响。五是国有企业改革进展缓慢，克难攻坚任务艰巨。化解矛盾的措施不够得力，不稳定因素依然存在。六是个别地方和部门抓落实不够，主动服务意识不强，办事效率低下，经济发展环境仍然不够宽松等。对此，我们一定高度重视，在今后的工作中认真加以克服和解决。

二、2008年经济社会发展总体要求和奋斗目标

今年是全面落实党的十七大精神的第一年和改革开放30周年，也是推动经济社会转入科学发展轨道的重要一年。当前，安阳的发展正处在承前启后、继往开来的关键时期，正处在厚积薄发、实现跨越的崭新阶段。随着国家促进中部崛起政策和我省率先实现中原崛起战略的实施，为我市带来了良好的发展机遇和环境。同时，中央加强宏观调控，提出了"两个防止"，严把"两个闸门"和"一个门槛"，也使我们面临着资金、土地、环境等多种因素制约。我们只有统一思想，凝聚力量，紧密结合安阳实际，扭住工业，抓住农业，大力发展第三产业，才能在竞相发展中赢得主动、抢占先机，才能在区域发展中率先突破、走在前列。

今年政府工作的总体要求是:以党的十七大精神为指导，全面落实科学发展观，进一步解放思想，转变观念，紧紧围绕全面建设小康社会和实现"一强五前"奋斗目标，深入实施"四路并进"发展战略，加快推进"三大转变"，大力推动主导产业优化升级，不断深化改革开放和自主创新，注重节能减排，着力改善民生，突出抓好重点项目建设、招商引资、县域经济和非公有制经济，努力保持经济社会又好又快发展。

今年我市经济社会发展的预期目标是:生产总值增长13%以上;财政一般预算收入增长15%以上;限额以上工业增加值增长19%;全社会固定资产投资增长20%;社会消费品零售总额增长15%;实际利用外资增长25%;居民消费价格总水平涨幅控制在低于上年实际水平;城镇居民人均可支配收入增长8%以上，农民人均纯收入增长7%以上;人口自然增长率控制在6‰以内;城镇登记失业率控制在4.5%以内，城镇新增就业人数6.5万人;单位生产总值能耗下降6%，化学需氧量、二氧化硫排放量分别下降6.26%和12.14%。

在实现上述目标的同时，市委、市政府今年还要全力为全市人民办好农民增收、安全饮水、医疗卫生、教育和社会保障等十个方面实事。这十个方面的实事已印发各位代表，请予审议。

三、2008年主要工作

完成今年的目标任务，实现经济社会又好又快跨越式发展，必须坚持高标准，明确新要求，谋求新突破，扎扎实实做好各项工作。

(一)加快推进"工业振兴工程"，大力实施项目带动主战略

坚持走新型工业化道路，紧紧扭住"工业振兴工程"这个经济发展的"牛鼻子"，着力优化工业结构，积极发展现代产业，促进工业由大变强。

(二)加快推进社会主义新农村建设，扎实做好"三农"工作

大力实施"四大兴农计划、三大惠民工程、十大农业产业链开发、十大农业增效技术和林业生态市建设"，加快向农业产业化强市迈进。

继续巩固和强化支农政策。加大国家粮食、农机、畜牧等直补政策和我市农业产业化、高效农业开发、林业"四荒"拍卖等以奖代补政策的落实，充分调动社会资金投向"三农"。

全力加强农业基础建设。加快落实上级支持资金，搞好彰武、汤河、石门等大中小型病险水库除险加固和漳南等灌区节水技改工程，抓好投资4000多万元的农田机井电力配套和13万亩水利示范方建设。

积极推进农业产业化。新增销售收入超亿元的龙头企业3-5家，培育市级以上龙头企业50家。继续培植农业产业化示范县、示范乡(镇)20个、大型农产品加工基地4个。进一步拉长十大农业产业链，搞好400万亩优质高产粮食基地建设。大力发展温棚瓜、果、菌、菜、花卉苗木等三种三收的优质高效农业，新增高效农业16万亩，总面积达到100万亩，新建各类示范园区45个。加快现代畜牧业发展，实施"552"畜牧业发展计划，新增畜禽养殖密集区78个、大中型规模养殖加工企业40家，全市畜牧密集区达到294个，大中型规模养殖加工企业达到184家。

全面启动林业生态市建设，打造绿色秀美安阳。通过进一步实施太行山绿化、防沙治沙和围城林、围村林、通道绿化等重点生态工程，力争经过几年努力，新增林地109万亩，使全市有林地面积达219万亩，林业年产值达36亿元。今年，在基本完成28.8万亩造林任务的同时，切实做好今冬明春30万亩造林规划，抓好万亩优质苗木基地建设。

切实加强新农村建设，不断改善农村生产生活条件。加强新农村建设规划，加大对农村公益事业的投入，今年市级财政安排700万元新农村建设资金，加快130个新农村试点村建设。建立和完善扶贫开发长效机制，争取国家资金2000万元，完成40个贫困村的整村推进和3.5万农民脱贫任务。以争取上级投入为主，解决安全饮水10万人。启动市级以奖代补资金，调动民间资金，完成自来水入户10万人。新增沼气用户4万户。完成中长期农村劳动力职业技能培训5万人。继续加大农林、畜禽防疫等支农服务体系建设，新建农村超市1000家。

(三)加快推进现代服务业，不断提高第三产业发展质量

推进服务业特别是现代服务业快速发展，今年市财政安排扶持资金1030万元，促进服务业发展速度与生产总值同步增长。

积极发展现代旅游业。大力实施文化旅游"22338"工程。分别将殷墟宫殿宗庙遗址、殷墟王陵遗址、羑里城捆绑和红旗渠青年洞、纪念馆、太行大峡谷捆绑，争创为国家5A级景区。加快马氏庄园创建4A级景区和周易文化申报国家非物质文化遗产的进程，做好内黄三杨庄汉代遗址保护展示馆等工程建设。
